# The Importance of Geriatric Care Models

**DOI:** 10.3390/geriatrics4010005

**Published:** 2018-12-27

**Authors:** James S. Powers

**Affiliations:** The Tennessee Valley Healthcare System Geriatrics Research, Education, and Clinical Center, Vanderbilt University School of Medicine, 7159 Vanderbilt Medical Center East, Nashville, TN 37232, USA; james.powers@vanderbilt.edu; Tel.: +1-615-936-3274; Fax: +1-615-936-3156

I am delighted to edit this Special Issue of *Geriatrics* focusing on Geriatric Care Models. With this volume describing 14 geriatric healthcare models, we showcase the importance of geriatric principles to healthcare systems worldwide. Healthcare is undergoing a value-based transformation. Value-driven healthcare strives to improve access to healthcare, to improve the patient’s experience and quality of care, and to moderate healthcare costs. Value-based purchasing drives quality metrics and can serve as an important lever for changes in healthcare delivery.

Geriatric patients consume a disproportionate share of healthcare resources. Estimates from the U.S. suggest that 50% of healthcare costs are attributed to 5% of the population characterized as high-risk, high-need patients [1]. The high-need population is characterized by heavy healthcare utilization and having functional self-care limitations. In addition to clinical needs, the high-need population also has behavioral, functional, and social needs [2].

Program targeting for added services for high-risk populations, and focusing service resources to needs, makes enormous sense. We are in the early stages of understanding how to identify appropriate patients and provide them the right intensity and mix of services. Innovative geriatric care models which demonstrate improved outcomes in health and well-being, care utilization, and cost moderation can be scaled to enhance care generally. Successful care models can involve the service setting, care delivery, and organizational culture. Focused management of high-need and frequent utilizers is critical and may include enhanced primary care, transitional and integrated care across settings, new techniques for patient monitoring, inter-professional team function, and continuous outcome assessment utilizing multiple data sources. The delivery transformation stimulated by value-based purchasing is forcing healthcare systems to manage populations and to evaluate the effects of different components of service along the continuum of care. 

Since 2010, the Centers for Medicare and Medicaid Services (CMS) has tested or announced over 30 alternative payment models in the U.S., developed in partnership with clinicians and designed to incentivize the delivery of high-quality patient care. Overall, these models are aligned with geriatric care principles [3]. Health systems must now respond to the challenges of a performance-based payment program that provides rewards for delivering well–coordinated, high-quality care with the help of technology.

The healthcare delivery and payment landscape is evolving rapidly, and these new models of healthcare delivery represent important changes that geriatricians have long supported. Many healthcare organizations are struggling to develop risk-based population health management strategies. Geriatric care models are focused on high-risk, high-need patients and can be valuable resources for healthcare organizations [4,5]. 

Included in this Special Issue on geriatric care are investigators and participants in innovative models, including advanced alternative payment models, transitions of care models, telehealth programs, patient-centered medical homes, acute care for elderly units, early mobility and healthy aging programs, medication reconciliation, and quality assessment and performance improvement (QAPI) programs. 

We dedicate this Special Issue to Kenneth Shay, DDS, MS who served as Chief of Geriatric Programs for the U.S. Department of Veterans Affairs from 2005 to 2018 and who worked tirelessly to foster the development of new healthcare models and testing of quality improvement innovations through the Department of Veterans Affairs Center of Excellence for Geriatrics, the Geriatric Research Education and Clinical Centers (GRECC). The Department of Veterans Affairs represents the largest U.S. integrated learning healthcare system.

With this Special Issue we are pleased to highlight innovative models of geriatric care from around the world in an effort to disseminate these impactful models promoting excellence in healthcare delivery.
